# A Case of Placenta Percreta Managed with Sequential Embolisation Procedures

**DOI:** 10.1155/2018/7213689

**Published:** 2018-03-15

**Authors:** Shannon Armstrong-Kempter, Supuni Kapurubandara, Brian Trudinger, Noel Young, Naim Arrage

**Affiliations:** ^1^Department of Women's and Newborn Health, Westmead Hospital, Westmead, NSW 2145, Australia; ^2^Western Sydney University, Campbelltown, NSW 2560, Australia; ^3^University of Sydney, Camperdown, NSW 2006, Australia; ^4^Sydney West Advanced Pelvic Surgery Unit, NSW, Australia; ^5^Department of Radiology, Westmead Hospital, Westmead, NSW 2145, Australia

## Abstract

**Background:**

The incidence of morbidly adherent placenta, including placenta percreta, has increased significantly over recent years due to rising caesarean section rates. Historically, abnormally invasive placenta has been managed with caesarean hysterectomy; however nonsurgical interventions such as uterine artery embolisation (UAE) are emerging as safe alternative management techniques. UAE can be utilised to decrease placental perfusion and encourage placental resorption, thereby reducing the risk of haemorrhage and other morbidities.

**Case:**

We describe one of the very few reported cases of placenta percreta which was successfully treated primarily with sequential artery embolisation. Our patient underwent four embolisation procedures over a period of 248 days, with no major morbidity or complications.

**Conclusion:**

Repeat UAE may be a beneficial primary management modality in cases of placenta percreta with bladder involvement.

## 1. Introduction

Placenta percreta is a serious obstetric complication where the placental villi penetrate through the myometrium into the uterine serosa and possibly adjacent organs. There are three degrees of morbidly adherent placenta (MAP): placenta accreta, increta, and percreta. Placenta percreta is the most severe but least common form of this condition, accounting for 7% of abnormally implanted placentas; however it is associated with a significantly higher maternal morbidity than the other varieties [1, 2]. The incidence of morbidly adherent placenta, including placenta percreta, has increased significantly over recent years, which has been attributed to increasing rates of caesarean delivery, although the mechanism remains speculative [3]. The most appropriate management for this life threatening condition is debated. We report a case that presented at the end of the first trimester with successful conservative management and detailed angiographic and ultrasound imaging.

## 2. Case Presentation

A 38-year-old female, G7P3 (three previous lower segment caesarian sections and 3 prior surgical terminations), presented to our hospital with a massive haemorrhage after surgical termination of pregnancy. The gestational age estimate was 14 weeks based on biparietal diameter on bedside ultrasound performed preoperatively at a private clinic. No formal ultrasound had been performed during her current pregnancy. A dilation of cervix and suction curettage were performed. Significant haemorrhage occurred with the loss exceeding 1000 ml. Syntocinon was administered intramuscularly and a Foley catheter was inserted into the uterus for tamponade. The patient was transferred from the clinic to a tertiary hospital.

**Figure 1:**
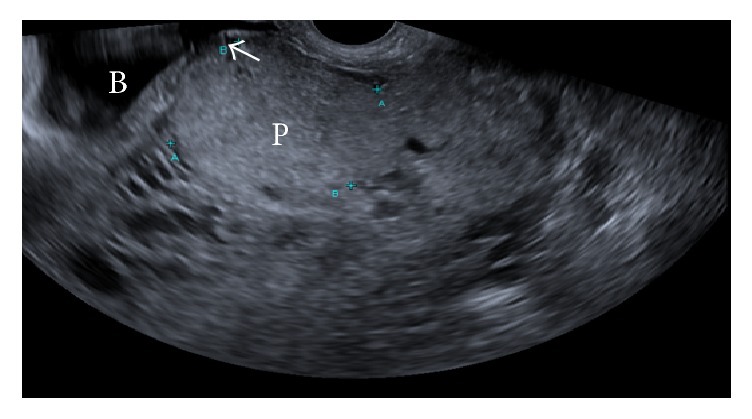
Retained placental tissue (P), 55 mm in diameter. Minimal myometrium noted between bladder (B) and placental tissue (arrowed).

**Figure 2:**
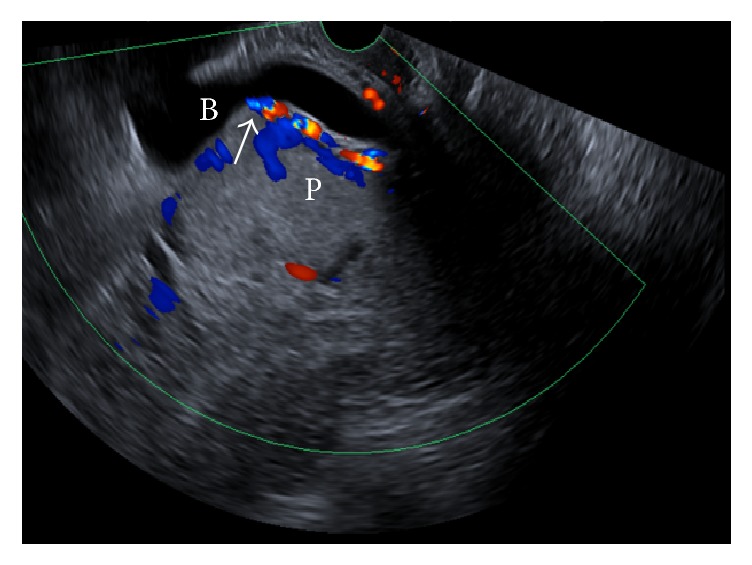
Large dilated vessels (arrowed) in the placental bed (P), possibly extending into bladder (B).

Upon arrival the patient was stable and there was no evidence of ongoing active blood loss. The patient was managed conservatively overnight after admission. A formal ultrasound scan performed the next day demonstrated retained placental tissue (with vascularity) at the site of previous caesarean scar, suggestive of a morbidly adherent placenta (see Figures 1 and 2).

Given the stability of the patient, she was offered the option of hysterectomy, placental resection, conservative management with arterial embolisation, or expectant management. The patient preferred to avoid a hysterectomy so as to preserve fertility as well as any other form of surgical intervention given the risk of significant surgical morbidity. A multidisciplinary discussion involving the treating team, urogynaecology, maternal-fetal medicine, and interventional radiology took place and together with the patient it was decided to proceed with arterial embolisation while leaving the placenta in situ. Given the lack of robust evidence with respect to embolisation and subsequent follow-up, it was decided that embolisation with very close outpatient monitoring would be the safest management option for this patient (see Table 1).

## 3. Discussion

The most appropriate management of placenta percreta with bladder involvement remains somewhat unclear. While a range of management options are presented in the literature, there is a lack of good quality data to indicate which management option is preferable, largely due to the paucity of such cases. These can be broadly categorised into hysterectomy with placenta in situ, placental resection, and conservative management modalities with or without a planned interval hysterectomy.

Conservative management, where the placenta is left in situ, can include expectant management, methotrexate administration, uterine artery embolisation (UAE), or a combination of these modalities. Conservative management offers the main advantages of minimising the risk of haemorrhage and other significant surgical morbidities at the time of delivery, as well as preserving fertility. One review (*n* = 407) found that 85.7% of women conceived following conservative treatment in all forms of morbidly adherent placenta (MAP); however in cases of placenta percreta specifically only 10% (1/10) had a subsequent pregnancy [4, 5]. It is also important to consider the significant recurrence risk of MAP, which has been reported to be as high as 29% [4]. Serious complications such as secondary haemorrhage, sepsis, and the need for emergency hysterectomy may occur with conservative management, and have been reported up to many months after delivery; thus this approach requires close surveillance.

In one of the largest case series of conservatively managed placenta percreta (*n* = 119), 61% of patients experienced at least one postoperative complication, compared to 12% in placental resection and caesarean hysterectomy groups [6]. The most common complications were emergency hysterectomy (50%) (even up to 9 months after caesarean section), haemorrhage (44%), sepsis (25%), and bladder injury (17%) [6]. Management with methotrexate has been described in some small case series and reports, with results ranging from successful placental resorption without complications [7–10] to significant complications including coagulopathy, haemorrhage, and need for secondary hysterectomy or placental removal [11–14]. Uterine artery embolisation has been used to manage placenta percreta primarily and in cases of postpartum haemorrhage; however a significant proportion of these (18–62%) may still require hysterectomy [5, 15–17]. For cases managed successfully with expectant management alone, reports of complete resorption range from 8 months to 3 years postpartum [18, 19]. It has been suggested by a number of case reports and series that leaving the placenta in situ at the time of delivery with a planned interval hysterectomy at a later date may be a safe management option, as there may be markedly decreased vascularity, allowing for technically easier hysterectomy with a reduced rate of peri- and postoperative complications [20–23]. However, this requires extensive planning and multidisciplinary input and there is insufficient consistent evidence to suggest an appropriate timeline before which a definitive interval hysterectomy should be offered. The unpredictability of complications with conservative management and associated morbidity necessitate taking a cautious, individualised approach with each case given the lack of robust evidence.

Local placental resection has also been presented as a conservative surgical alternative in cases of placenta percreta with bladder involvement; however there have been mixed results depending on the resection method utilised. In one prospective study (*n* = 68), local resection was performed via retrovesical and parametrial dissection with subsequent repair of the anterior wall defect, with 26% of patients requiring secondary hysterectomy, the majority due to extensive uterine destruction, and two cases of inadvertent ureteric ligation [24]. In a retrospective review of local resection (*n* = 17), there were no reported cases of urological complications and only two cases of haemorrhage, neither requiring hysterectomy [6]. One small cohort study (*n* = 19) has proposed a method of local resection involving myometrial excision leaving the area of placental involvement of the bladder intact and uterine artery balloon occlusion, which has shown a reduced rate of postpartum haemorrhage, secondary hysterectomy, and duration of hospital stay when compared to leaving the placenta in situ [25, 26]. A systematic review found that partial resection resulted in a subsequent pregnancy in 19/26 (73%) cases of morbidly adherent placenta [5].

Caesarean hysterectomy has historically been the treatment of choice for abnormally invasive placenta, where the placenta along with the uterus is removed at the time of delivery. This minimises the risk of long term complications, such as sepsis, haemorrhage, and need for emergency hysterectomy. However, there is considerable morbidity associated with this procedure, with significant intraoperative and postoperatively complications, including maternal death (up to 5%) [27]. Urology involvement preoperatively has been shown to reduce the rates of urological complications [28]. A large retrospective review (*n* = 66) found that 30% of cases managed with caesarean hysterectomy resulted in some form of complication, with 17% suffering a bladder injury and 7.6% postoperative haemorrhage [6]. Conversely, one prospective case series (*n* = 58) had only 6.9% of patients enduring a bladder injury and 1.7% requiring reoperation due to haemorrhage; however, almost one-third (29.3%) received more than 4 units of red blood cells [29]. There have been a number of case reports presenting modified caesarean hysterectomy methods, with techniques such as intentional cystotomy and resection of the affected bladder wall with subsequent bladder repair [30], subtotal hysterectomy with invasive portion of placenta left in situ [31], and retrograde caesarean hysterectomy [32]. These methods have been proposed in an attempt to minimise urological complications and intraoperative blood loss; however they lack sufficient supporting evidence and do not seem to result in a significant reduction of morbidity. Caesarean hysterectomy in conjunction with arterial embolisation and/or arterial balloon occlusion has been shown to reduce intraoperative blood loss and transfusion requirements when compared to caesarean hysterectomy alone [6, 33, 34].

This is one of the first reported cases of serial embolisation for the primary management of placenta percreta. While there have been other case reports of sequential arterial embolisation, to our knowledge, this is the first report of so many embolisation procedures, utilised as the only management method [8, 35]. Given the fact that our patient did not have a progressing pregnancy and that she had completed her family but was eager for uterine conservation and also very compliant, we were able to try this uterine sparing method. In a retrospective review including nine cases managed with primary arterial embolisation, 78% (7/9) did not experience major morbidity, with only two requiring hysterectomy [36] and resorption of placental tissue has been reported after 4–6 months with no complications [16, 37–39]. However, there may be significant adverse outcomes associated with this management option, including secondary PPH, DIC, and emergency hysterectomy [36, 40, 41]. There may also be adverse outcomes resulting directly from arterial embolisation, including postembolisation syndrome, uterine scarring, endometrial atrophy, and secondary amenorrhoea [42, 43]. One systematic review found that arterial embolisation resulted in a subsequent menstruation in 6/10 (60%) but subsequent pregnancy was not reported in any (0/7) cases, which is significantly lower than other forms of conservative management or partial resection and may be associated with significant increased risk [5]. Our patient had a successful outcome, given we were able to conserve the uterus and achieve regression of placental tissue without encountering any significant morbidity during the process. There was a significant risk of life threatening haemorrhage as with any case of conservatively managed placenta percreta. This risk was further increased given the extensive blood supply to the retained placental tissue identified in initial angiogram, which took four embolisation procedures to adequately devascularise. The treatment took nine months and required extensive monitoring and intervention; however this is within the range of 4–12 months as reported by other similar case studies in the literature [5, 8, 35, 37, 38].

## 4. Conclusion

While the incidence of morbidly adherent placenta, including placenta percreta, is sure to increase in the years to come, there is a lack of robust evidence regarding the most appropriate management; thus management must be individualised. We present a case of a successfully managed placenta percreta with serial arterial embolisation procedures over a period of nine months, which resulted in placental regression and uterine preservation without significant morbidity.

## Figures and Tables

**Figure 3:**
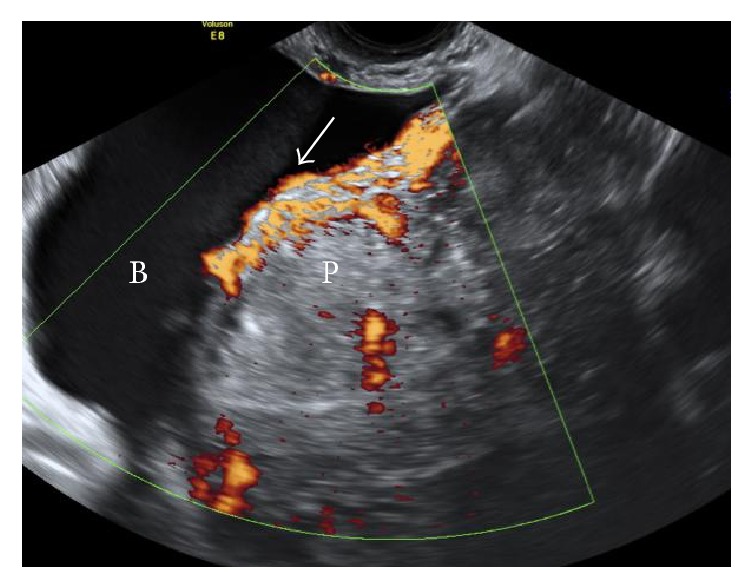
Ongoing evidence of adherent placenta (P) with likely bladder (B) invasion (arrowed) and dilated vesical and uterine vessels.

**Figure 4:**
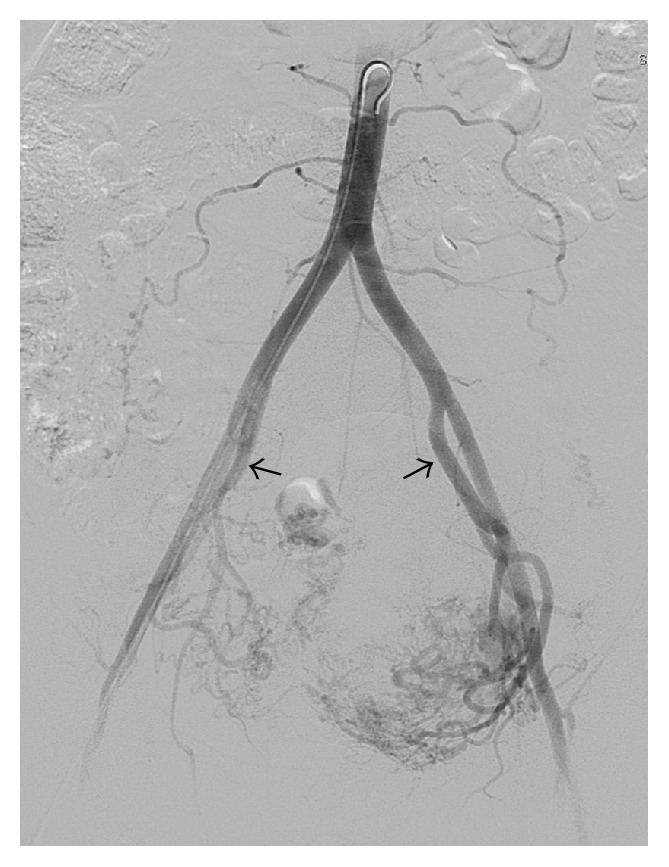
Pelvic angiogram—early arterial phase. Both internal iliac arteries (arrowed) show extensive abnormal arterial supply to the uterus, more evident on the left.

**Figure 5:**
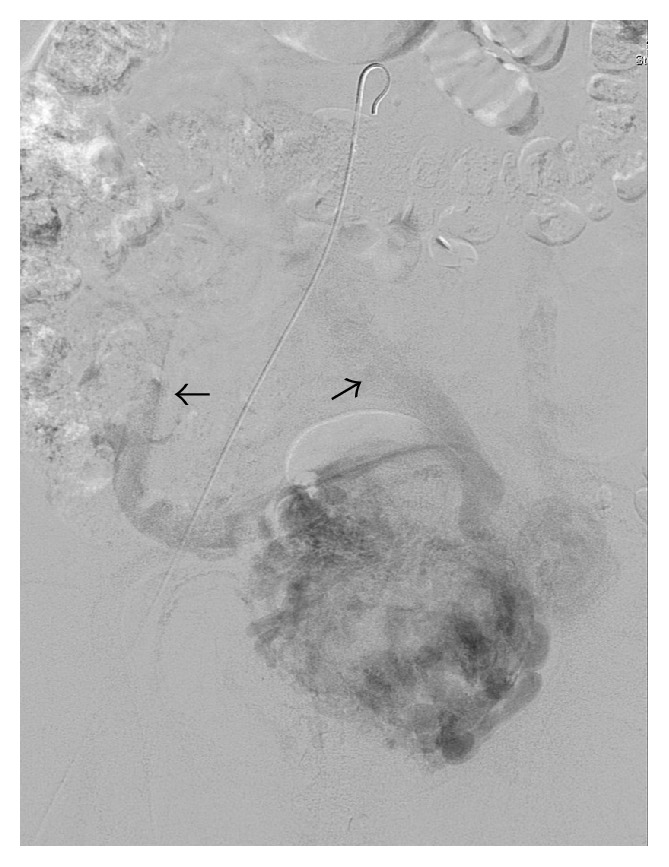
Pelvic angiogram—late venous phase. Early venous drainage to internal iliac veins (arrowed).

**Figure 6:**
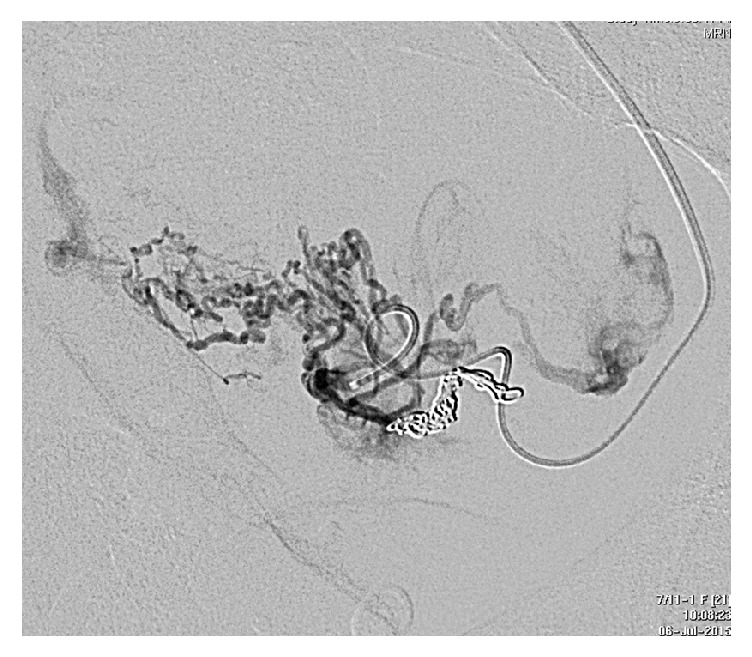
Microcatheter metal coil embolisation of the abnormal left internal iliac arteries.

**Figure 7:**
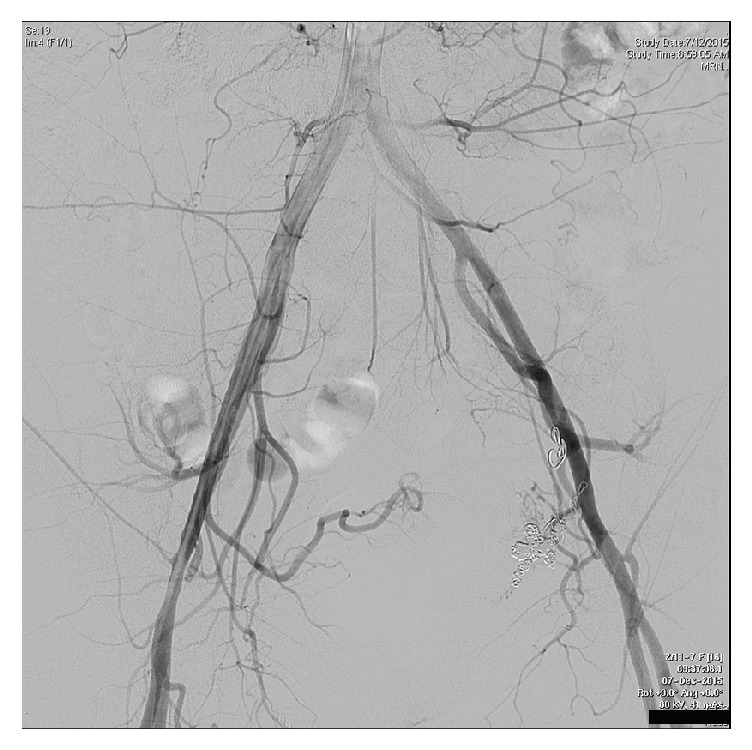
Pelvic angiogram following serial embolisation shows no persisting abnormal uterine vessels.

**Figure 8:**
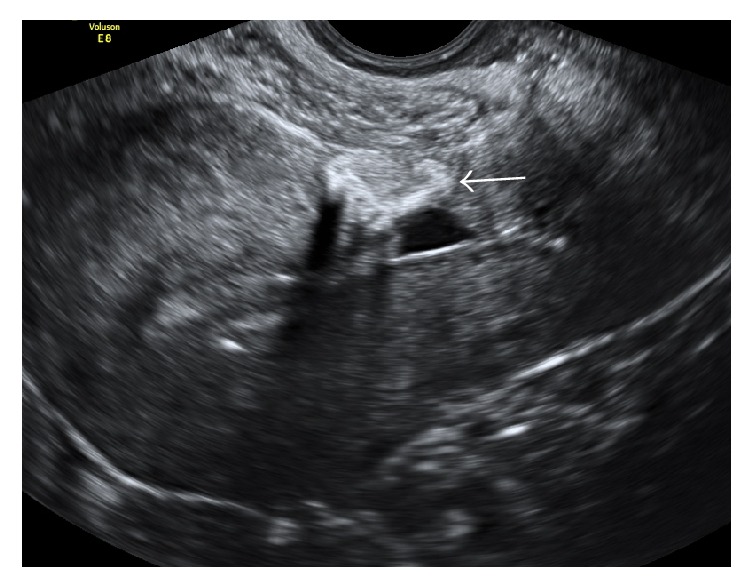
Avascular echogenic mass (16 × 15 × 10 mm) at the site of caesarean scar (arrowed).

**Figure 9:**
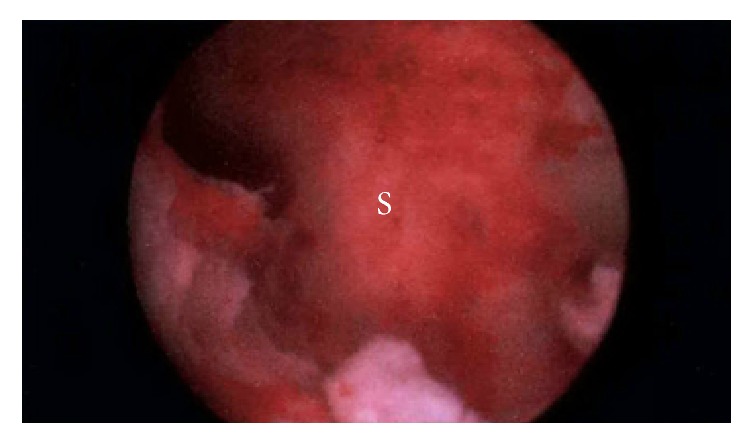
Uterine septum (S). Otherwise normal uterine cavity.

**Table 1:** A timeline summary of the management of this patient.

Days after surgical uterine evacuation	Events and Images
Day 22	(i) Ultrasound showed persistent retained placental tissue with significant vascularity and extension into bladder with no overlying myometrium, suggestive of placenta percreta with bladder involvement
	(See Figure 3)
	(ii) Multidisciplinary discussion between gynaecologist, urogynaecologist, maternal fetal medicine specialist, and patient
	(iii) The options of management discussed included expectant management, abdominal hysterectomy, or uterine artery embolisation
	(iv) Uterine artery embolisation was decided

Day 33	(i) Initial angiogram showed very large, tortuous, abnormal uterine arteries, particularly on the left side; thus it was decided to proceed with initial embolisation with the view that multiple procedures would be required to adequately devascularise the retained placental tissue
	(ii) This decision was based on attempting to minimise undue ischemia and pain to the patient, and therein any hospital admissions, as well as minimising the radiation exposure to this young patient by spreading the embolisation over multiple session
	(iii) Left sided arterial embolisation performed via microcatheter, using Boston Scientific Helical pushable metal coils (4 mm + 6 mm) and Boston scientific contour embolisation particles (250–350 microns)
	(See Figures 4, 5, and 6)

Day 36	(i) Ultrasound showed persistence of retained placental tissue with significant vascularity

Day 54	(i) Pelvic angiogram showed persistent uterine vascular abnormality with some regression since the initial embolisation procedure
	(ii) Further embolisation of two arterial branches of the left internal iliac artery
	(iii) Regression of persistent PV bleeding and return of regular menses

Day 57	(i) Serum beta HCG 7

Day 107	(i) Angiogram showed further improvement of the uterine vascular abnormality
	(ii) Further embolisation of a branch of the right internal iliac artery

Day 177	(i) Pelvic angiogram showed a single abnormal feeding vessel to the vascular anomaly off the right internal iliac artery, which was successfully embolised
	(ii) No further abnormal vessels, including intraperitoneal feeding vessels, were identified
	(See Figure 7)

Day 241	(i) Ultrasound showed persistent uterine mass (16 × 15 × 10 mm); however this was avascular and significantly reduced in size as compared to earlier ultrasound images
	(See Figure 8)

Day 248	(i) Hysteroscopy was performed which showed no evidence of residual placental tissue over the anterior uterine wall. Endometrium overlying possible remnant placental tissues could not be ruled out. A uterine septum was identified which was divided with scissors
	(See Figure 9)

Day 283	(i) Patient was well, continuing to have regular menstrual periods with no abnormal bleeding
